# Optimal vaccination age varies across countries

**DOI:** 10.1073/pnas.2105987118

**Published:** 2021-07-06

**Authors:** Héctor Pifarré i Arolas, Enrique Acosta, Mikko Myrskylä

**Affiliations:** ^a^Centre for Research in Health Economics, Universitat Pompeu Fabra, 08002 Barcelona, Spain;; ^b^Max Planck Institute for Demographic Research, 18057 Rostock, Germany;; ^c^Center for Social Data Science, University of Helsinki, 00014 Helsinki, Finland

Who should be vaccinated first against COVID-19? This complex question links science, policy, and ethics, and was recently tackled by Goldstein et al. (1) (henceforth GCW). GCW offer an advance that reduces the complexity of this issue by showing that, under certain conditions, vaccinating the oldest first maximizes both the number of deaths averted and the expected number of life years saved (eYLL). GCW demonstrate their point using high-quality data for the United States, Germany, and South Korea. Their result, to the extent it holds more generally, solves the ethical concern of a trade-off between saving lives versus life years.

GCW’s finding is important beyond the COVID-19 pandemic. Within a large class of mortality models and assuming that mortality from a specific condition is proportional to all-cause mortality, their insight applies to many prioritization problems where treatment must be rationed. Proportionality may hold for conditions in which mortality is primarily driven by age-related frailty. Yet, conditions driven by behavior or risk taking may follow a different age profile and require targeting of younger ages.

How well does the proportionality assumption, and the finding that vaccinating the oldest saves the most life years, hold for COVID-19? We studied this in 40 countries with data on confirmed COVID-19 deaths (2, 3) as of January 15, 2021, before widespread vaccination. For illustration purposes, Fig. 1*A* plots COVID-19 and all-cause mortality by age for three selected countries, suggesting deviations from proportionality. Red lines represent the age-specific minimum hypothetical mortality such that vaccinating the given age group would save as many eYLL as vaccinating the oldest. Only when this (red) rate is above the (black) observed mortality does vaccinating the oldest maximize eYLL. This pattern holds for the United States, but not for Peru, where the lines cross over at age 55 years, or Chile (at 75 years). For the United States, we replicate GCW’s finding of monotonically increasing eYLL by vaccination age (Fig. 1*B*); in Chile and Peru, the eYLL curve peaks at ages 90 and 70 years, respectively. Deviations from monotonic increase hold also if calculations are based on excess mortality (in green).

**Fig. 1.**
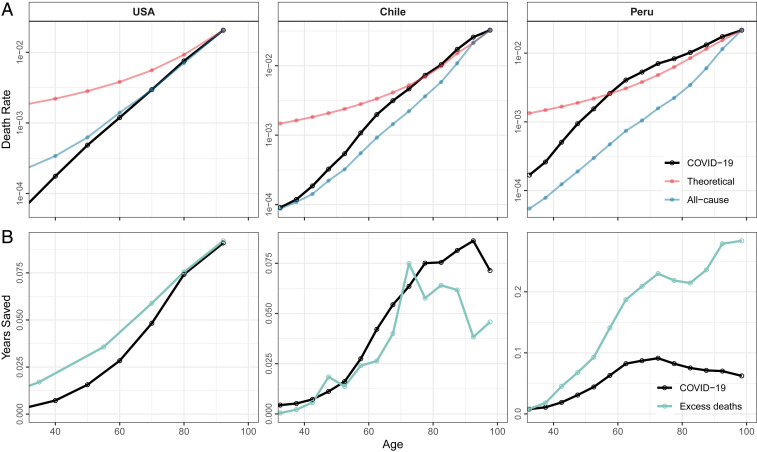
Age pattern of COVID-19 mortality and potential life years saved in the United States, Chile, and Peru. *A* displays, in log scale, the age patterns of COVID-19 death rates (black line); the slope of all-cause death rates, normalized to intercept the COVID-19 death rate in the last age group (blue line); and the theoretical age-specific COVID-19 death rate that equalizes eYLL with the age group with the highest COVID-19 death rates (red line). *B* depicts the person-years saved per effective vaccination at each age based on confirmed COVID-19 deaths (black line) and on excess mortality estimates (green line).

Fig. 2 shows crossover ages for all 40 countries. For 28/40 countries, this is the oldest. For 12/40 countries, crossover occurs at a (sometimes substantially) younger age, implying that a vaccination criterion based on eYLL does not prioritize the oldest. These results are qualitatively robust to alternative calculations of life expectancy in the last age group, and to replacing COVID-19 deaths with excess mortality (4), even though optimal ages and the exact number of countries deviating from the “oldest rule” vary.

**Fig. 2.**
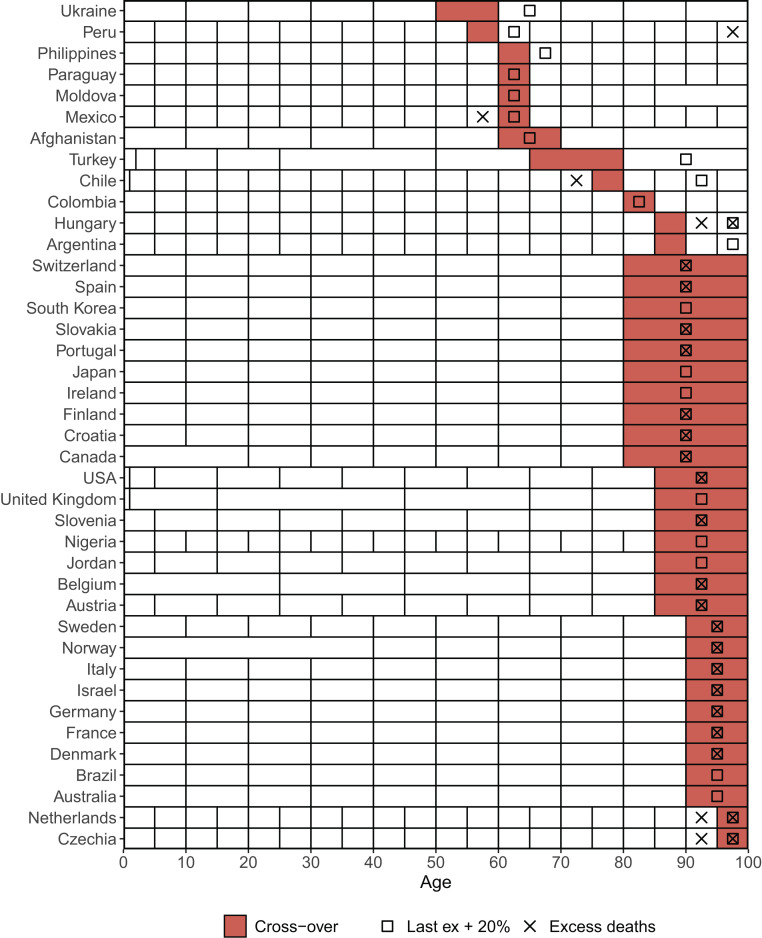
Age group breakdown of COVID-19 death reports by country. The first age at which we find a crossover is shown in red. Squares indicate the crossover age groups at which the last age group’s remaining life expectancy was increased by 20%. This crossover marks the youngest age at which vaccination would save as many life years as vaccination in the oldest age group. Crosses represent the crossover age groups computed using excess mortality instead of official COVID-19 death counts. The code and data for replication are available at our repository at https://osf.io/qnpm7.

While GCW’s demographic insight applies to many countries and, in these contexts, helps to resolve the dilemma between saving lives versus life years, the pattern is not universal, as there remains heterogeneity in the vaccination age that maximizes eYLL. This is consistent with studies showing that deaths and life years lost are higher at younger ages in low- and middle-income economies than in high-income countries (5–7). Decision makers may still need to address troubling ethical questions around vaccination plans.

## Data Availability

The code and data for replication are available at our Open Science Framework repository: https://osf.io/qnpm7 (8).
